# Donor type dynamics and associated factors among blood donors in northern Ghana: a six-year retrospective analysis

**DOI:** 10.3389/fepid.2026.1905551

**Published:** 2026-09-08

**Authors:** Yula Salifu, Prosper Gyebuni, Torjim Salifu, Eleonora B. Wobi, Joseph Lasong

**Affiliations:** Department of Population, Family and Reproductive Health, School of Public Health, University for Development Studies, Tamale, Ghana

**Keywords:** blood donors, blood safety, donation frequency, first-time donors, Ghana, repeat donors

## Abstract

**Background:**

Repeat blood donors have lower transfusion-transmissible infection (TTI) rates, making donor type distribution critical for blood safety. This study characterised donor type patterns, factors associated with first-time donation, and TTI prevalence differences in Northern Ghana.

**Methods:**

A retrospective cross-sectional analysis of 3,546 donor records (2017–2022) from seven health facilities in Upper West, Northern, and Oti regions. Donor type (first-time vs. repeat) was the primary outcome. Descriptive statistics, chi-square tests, and multivariable logistic regression were used. TTI prevalence, defined as positivity for at least one of HIV, HBV, HCV, or syphilis (“any TTI”), was compared between donor types. Significance was set at *α* = 0.05.

**Results:**

First-time donors constituted 82.0% (2,907) and repeat donors 18.0% (639). Repeat donation increased from 12.3% (2020) to 22.6% (2022). Rural donors (93.1% first-time) and non-volunteer donors (82.5% first-time) showed higher proportions of first-time donation (both *p* < 0.001). In adjusted analysis, older age (AOR 0.97 per year) and urban residence (AOR 0.69) were associated with lower odds of being first-time. The Oti region had markedly higher odds (AOR 491.65). First-time donors had significantly higher TTI prevalence: any TTI (16.06% vs. 5.48%), HBV (5.64% vs. 1.88%), HCV (2.51% vs. 0.16%), and syphilis (8.67% vs. 3.60%).

**Conclusions:**

The predominance of first-time donors (82%) and low repeat donation rates represent a blood safety vulnerability that would benefit from targeted donor retention programs, particularly in rural areas and the Oti region.

## 1 Introduction

The safety and adequacy of blood supply depend on a stable base of voluntary, repeat blood donors (1). Repeat donors: individuals who have donated two or more times, are considered central to blood safety because they undergo repeated screening, receive ongoing health education, and are more likely to be motivated by altruism rather than external pressure (2). In contrast, first-time donors, particularly those donating for replacement or family reasons, have consistently been shown to have higher prevalence of transfusion-transmissible infections (TTIs) across multiple settings (3, 4).

The World Health Organization (WHO) recommends that all countries transition to 100% voluntary non-remunerated blood donation (VNRD) as a key blood safety strategy (1). However, many sub-Saharan African countries, including Ghana, continue to rely heavily on replacement and family donations, with voluntary and repeat donation rates remaining low (5). Understanding the factors that distinguish first-time from repeat donors is essential for designing effective donor recruitment and retention programs.

In Ghana, limited data exist on donor type dynamics, particularly in the northern regions, which have historically received comparatively less programmatic investment in blood-safety infrastructure than southern regions of the country. This is a particular concern given that Ghana carries a substantial burden of transfusion-transmissible infections, with national hepatitis B prevalence historically among the highest in the sub-region (6) and a continuing HIV burden nationally (7). The few available studies have focused primarily on TTI prevalence rather than donor type characteristics. This knowledge gap hampers efforts to implement targeted interventions for donor conversion and retention.

This analysis aimed to: (i) determine the proportion of first-time vs. repeat donors overall and by key demographic and geographic characteristics; (ii) identify factors independently associated with being a first-time donor; (iii) compare TTI prevalence between first-time and repeat donors; and (iv) examine temporal trends in donor type distribution from 2017 to 2022.

## 2 Materials and methods

### 2.1 Study design and setting

This study was a retrospective cross-sectional analysis of blood donor records from seven health facilities located in three regions of Northern Ghana: Upper West, Northern, and Oti. The study period covered six consecutive years, from 1 January 2017 to 31 December 2022. The facilities included:

- **Upper West Region:** Wa Municipal Hospital.
- **Northern Region:** Tamale Central Hospital, Tamale West Hospital, SDA Hospital Tamale.
- **Oti Region:** Nkwanta South Municipal Hospital.
- **Other region (Bono East):** Holy Family Hospital, Techiman (categorised as “Other” for analysis).

These facilities were selected because they are the primary blood collection centres serving their respective districts and perform routine donor screening and documentation according to national guidelines (2). The study was conducted in accordance with the Strengthening the Reporting of Observational Studies in Epidemiology (STROBE) guidelines for cross-sectional studies (8).

### 2.2 Blood collection and donor screening procedures

#### 2.2.1 Donor recruitment and selection

Blood donors were recruited through two main pathways: (1) voluntary non-remunerated donation (mobile clinic drives, school-based programs, workplace campaigns, and community outreach) and (2) replacement/family donation (requested by a patient's relatives). All potential donors underwent a standardised pre-donation assessment following the National Blood Service Ghana protocol (2). This included:

- Registration and demographic data collection: Age, sex, residential address (urban/rural, classified by Ghana Statistical Service definitions), and region.
- Medical history questionnaire: A structured interview covering risk factors for TTIs (e.g., history of jaundice, sexually transmitted infections, surgery, tattoos, blood transfusion, and high-risk behaviours). Donors with any positive response on deferrable criteria (e.g., recent high-risk exposure, symptoms of infection) were temporarily or permanently deferred.
- Physical examination: Vital signs (blood pressure, pulse, temperature), body weight (minimum 50 kg, per national protocol), and general physical assessment to ensure fitness for donation. Donors with haemoglobin <12.5 g/dL (for females) or <13.0 g/dL (for males) were deferred. Body weight was applied only as a pass/fail eligibility screen at the point of donation and was not captured as a variable in the analytic dataset; it therefore does not appear as a covariate in subsequent analyses.

#### 2.2.2 Blood collection procedure

Eligible donors underwent venepuncture of the antecubital vein under aseptic conditions. A standard 450 mL whole blood unit was collected into sterile triple blood bags containing citrate-phosphate-dextrose (CPD) anticoagulant. Collection was performed by trained phlebotomists or nurses following national safety guidelines. Post-donation care included observation for 15 min, refreshments, and iron supplementation advice.

#### 2.2.3 Post-collection testing

Blood samples were tested for TTIs at each facility's laboratory using rapid diagnostic tests (RDTs) following manufacturer instructions and national algorithms (2):

- **HIV:** Determine™ HIV-1/2 (Alere Medical Co., Japan) and Uni-Gold™ (Trinity Biotech, Ireland) in parallel; discordant results resolved by Western blot (only when clinically indicated).
- **HBV (HBsAg):** SD Bioline HBsAg (Standard Diagnostics, Korea).
- **HCV (anti-HCV):** SD Bioline HCV (Standard Diagnostics, Korea).
- **Syphilis (anti-treponemal):** SD Bioline Syphilis 3.0 (Standard Diagnostics, Korea).

All positive results were confirmed by a second RDT of a different brand; only repeatedly reactive units were discarded. Donors with confirmed TTIs were referred for clinical management according to national guidelines. All negative units were labelled and stored at 2–6 °C for transfusion.

### 2.3 Study population and sampling strategy

#### 2.3.1 Target population

All individuals who donated blood at the seven participating facilities between 1 January 2017 and 31 December 2022 and had complete records for donor type classification, demographic data, and TTI test results.

#### 2.3.2 Sampling method

We employed a complete enumeration (census) approach. All available donor records from the seven facilities during the study period were extracted, provided they met the following inclusion criteria:

- Donation date between 1 January 2017 and 31 December 2022.
- Complete documentation of donor type (first-time or repeat, based on facility history).
- Age between 16 and 70 years (legal donation age in Ghana).
- Complete results for HIV, HBV, HCV, and syphilis testing.
- No missing data on residence, donation purpose, or region.

#### 2.3.3 Exclusion criteria

Records were excluded if:

- Donor age was outside 16–70 years.
- Any TTI test result was missing or invalid.
- Donor type could not be determined (e.g., missing previous donation history).
- Donation purpose (volunteer/non-volunteer) was not recorded.

#### 2.3.4 Record screening process

A total of [*n* = 4,032] donor records across the seven facilities were initially reviewed for the study period. Records were sequentially excluded for: age outside the 16–70-year range (*n* = 57); missing or invalid TTI results (*n* = 241); indeterminate donor-type classification (*n* = 76); and missing residence, purpose, or region data (*n* = 112), yielding a final analytic sample of 3,546 records.

### 2.4 Variables and operational definitions

#### 2.4.1 Primary outcome; donor type

- First-time donor: An individual with no documented record of prior blood donation at the same facility before the index donation. This classification relied on the facility's donor register, which maintained a unique identifier (usually name and date of birth or national ID) for each donor. Donors were only classified as first-time if the facility's records showed zero previous donations.
- Repeat donor: An individual with two or more documented donations at the same facility, including the index donation. The number of previous donations was not always available; therefore, repeat was defined as any donation that was not the first at that facility.

#### 2.4.2 Important limitation

This classification does not capture donations made at other facilities. A donor classified as “first-time” may have donated elsewhere, leading to misclassification bias (9).

#### 2.4.3 Donation purpose (voluntary status)

- Volunteer donor (voluntary non-remunerated): A donor who gave blood of their own free will without receiving money or other payment, and who was not directed to donate to a specific patient. This category included donors from mobile drives, school campaigns, workplace drives, and community outreach.
- Non-volunteer donor: A donor who gave blood in response to a specific request for a named patient (replacement donation), or who was a family member, friend, or neighbour of a patient. This category includes “family/replacement” and “directed” donations as defined by WHO (1).

#### 2.4.4 Residential status

- Urban: Donor resided in a municipal or district capital as defined by the Ghana Statistical Service (10).
- Rural: All other locations (villages, hamlets, and settlements not designated as urban).

Age: Donor age in completed years at the time of donation, calculated from date of birth (if available) or reported age. Age was analysed both as a continuous variable (centred at the mean) and categorised into five groups (16–25, 26–35, 36–45, 46–55, 56–70) based on WHO classification for adult populations (1).

Region: Derived from the facility location where the donation occurred. Categories: Northern (Tamale Central Hospital, Tamale West Hospital, SDA Hospital Tamale); Upper West (Wa Municipal Hospital); Oti (Nkwanta South Municipal Hospital); Other (Holy Family Hospital, Techiman, Bono East Region).

Blood group (ABO and Rh): Determined by standard tube agglutination using monoclonal antisera (Anti-A, Anti-B, Anti-D) (11). ABO groups were categorised as A, B, AB, or O. Rh was categorised as positive or negative.

#### 2.4.5 TTI status

- HIV positive: Reactive on both initial and confirmatory RDT.
- HBV positive: Reactive for HBsAg.
- HCV positive: Reactive for anti-HCV antibodies.
- Syphilis positive: Reactive for anti-treponemal antibodies.
- Any TTI: Positive for at least one of the four infections above (HIV, HBV, HCV, or syphilis).

### 2.5 Data extraction and management

Data were extracted from paper-based and electronic donor registers at each facility by trained research assistants (two MPH holders). A standardised data extraction form was used to collect: donor identification number (anonymised after extraction); date of donation; donor type (first-time/repeat); age, sex, residence, region; donation purpose (volunteer/non-volunteer); ABO and Rh blood group; and TTI test results (HIV, HBV, HCV, syphilis).

Data were double-entered into Microsoft Excel (2016) by two independent data entry clerks. Discrepancies were resolved by referring to the original records. After cleaning and validation, the dataset was exported to R version 4.5.3 for statistical analysis. No donor-identifying information (names, addresses, phone numbers) was retained in the analytical dataset.

#### 2.5.1 Missing data handling

Variables with missingness >5% were excluded from multivariable analysis. In this dataset, missingness was <1% for all variables except donation purpose (0.2%). For the few records with missing donation purpose (*n* = 8), we imputed using the mode of the facility-year strata. A complete-case sensitivity analysis gave nearly identical results (not shown); we therefore present the imputed dataset.

### 2.6 Statistical analysis

All analyses were performed using R version 4.5.3 (R Foundation for Statistical Computing, Vienna, Austria). The significance level was set at *α* = 0.05 (two-tailed).

#### 2.6.1 Descriptive analysis

Frequencies and percentages were calculated for donor type overall and stratified by region, residence, donation purpose, age group, sex, blood group, and Rh status. 95% confidence intervals for proportions were calculated using the Wilson score method (for comparisons with external benchmarks) but are not reported in tables for brevity; they are available upon request.

#### 2.6.2 Bivariate analysis

Associations between each categorical variable and donor type (first-time vs. repeat) were assessed using Pearson's chi-square test. For 2 × 2 tables with expected cell counts <5, Fisher's exact test was used. For larger tables with sparse cells, *p*-values were estimated using Monte Carlo simulation with 2,000 replicates (12). Results are presented with *χ*^2^ statistics and *p*-values.

#### 2.6.3 Logistic regression analysis

Univariate logistic regression was performed to estimate crude odds ratios (OR) and 95% confidence intervals (CI) for each potential predictor of being a first-time donor. Variables with *p* < 0.20 in univariate analysis were considered candidates for the multivariable model (13). Multivariable logistic regression was fitted using the following *a priori* selected variables: sex, age (centred), residence, donation purpose, and region (Northern as reference). Blood group and Rh status were examined only in univariate analysis and were not entered into the multivariable model; they were included as exploratory covariates because (a) ABO/Rh distribution can differ across facility catchment populations and may act as a marker of geographic sub-population structure relevant to donor-type composition, and (b) prior sub-Saharan African donor studies have reported associations between ABO group and both donor-return behaviour and TTI risk (14), providing an empirical rationale for testing the association descriptively here. This analysis is exploratory and hypothesis-generating rather than confirmatory, and was not adjusted for multiple comparisons.

The final multivariable model was built using manual stepwise backward elimination (likelihood ratio test criterion), retaining variables with *p* < 0.05 or those considered clinically important (e.g., age), consistent with established approaches to confounder retention in multivariable modelling (15, 16). Adjusted odds ratios (AOR) with 95% CI were reported. Model fit was assessed using the Hosmer-Lemeshow goodness-of-fit test (13); a *p*-value >0.05 indicates acceptable fit. The model's discrimination was evaluated using the area under the receiver operating characteristic curve (AUC); an AUC of 0.72 was obtained, indicating reasonable discrimination.

#### 2.6.4 Collinearity diagnostics

Variance inflation factors (VIF) were calculated for all continuous and categorical predictors. No VIF exceeded 2.5, indicating no problematic collinearity. Sparse-data sensitivity check: because the Oti region contained only three repeat-donation events among 1,512 donors, the corresponding univariate odds ratio was recomputed using Firth's penalized-likelihood logistic regression (17), a bias-reduction approach recommended for sparse or near-separated binary data (18), and compared with the standard maximum-likelihood estimate; results are reported in Section 4.9.

#### 2.6.5 TTI comparison

Prevalence of each TTI and “any TTI” (positive for at least one of HIV, HBV, HCV, or syphilis) was calculated separately for first-time and repeat donors. Differences were tested using chi-square tests (or Fisher's exact for HIV, where repeat donors had zero events).

#### 2.6.6 Temporal trend analysis

The proportion of repeat donors was plotted by calendar year. No formal time-series modelling was performed due to the small number of years; trends are described narratively.

No *post-hoc* analyses were conducted.

### 2.7 Ethical considerations

Ethical approval was obtained from the University for Development Studies Ethics Review Board (approval number UDS/RB/date: 01/01/22). Permission to access donor records was granted by each participating health facility's management and the respective regional health directorates. Because the study involved only retrospective, de-identified routine programme data, the requirement for individual informed consent was formally waived by the ethics board, consistent with Ghana Health Service guidelines for secondary data use (19). All data were anonymised prior to analysis; facility names were retained only for regional classification. No donor-identifying information (names, national ID numbers, phone numbers, or exact addresses) was extracted or stored. The study was conducted in accordance with the Declaration of Helsinki (20) and Ghana's national guidelines for health research using secondary data (19). The STROBE checklist for cross-sectional studies was followed (8).

## 3 Results

### 3.1 Overall donor type distribution

Among 3,546 donors included in the analysis, 2,907 (82.0%) were first-time donors and 639 (18.0%) were repeat donors (Supplementary Table S1).

### 3.2 Donor type by demographic, geographic, and donation characteristics

Table 1 presents the distribution of donor type by region, residence, donation purpose, age group, and sex.

**Table 1:** Donor type distribution by demographic, geographic, and donation characteristics.

Variable	Total *N*	First-time *N* (%)	Repeat *N* (%)	*P*-value
Region				<0.001
Oti	1,512	1,509 (99.8)	3 (0.2)	
Upper West	1,313	975 (74.3)	338 (25.7)	
Northern	542	380 (70.1)	162 (29.9)	
Other	179	43 (24.0)	136 (76.0)	
Residence				<0.001
Rural	1,755	1,634 (93.1)	121 (6.9)	
Urban	1,708	1,193 (69.8)	515 (30.2)	
Donation purpose				<0.001
Non-volunteer	3,169	2,616 (82.5)	553 (17.5)	
Volunteer	183	100 (54.6)	83 (45.4)	
Age group (years)				0.504
16–25	1,457	1,302 (89.4)	155 (10.6)	
26–35	1,329	1,027 (77.3)	302 (22.7)	
36–45	614	471 (76.7)	143 (23.3)	
46–55	136	99 (72.8)	37 (27.2)	
56–70	8	6 (75.0)	2 (25.0)	
Sex				<0.001
Female	311	191 (61.4)	120 (38.6)	
Male	3,234	2,715 (84.0)	519 (16.0)	

Authors' analysis of blood donor records, 2017–2022.

Donor type varied by region (*χ*^2^ = 215.53, *p* < 0.001): the Oti region had 99.8% (1,509/1,512) first-time donors, Upper West had 74.3% (975/1,313), Northern had 70.1% (380/542), and the “Other” region had 24.0% (43/179).

Rural donors (*n* = 1,755) were 93.1% first-time vs. 69.8% among urban donors (*n* = 1,708) (*χ*^2^ = 114.17, *p* < 0.001).

Non-volunteer donors (*n* = 3,169) were 82.5% first-time vs. 54.6% among volunteer donors (*n* = 183) (*χ*^2^ = 34.55, *p* < 0.001).

Across age groups, the proportion of first-time donors was 89.4% (16–25 years), 77.3% (26–35 years), 76.7% (36–45 years), 72.8% (46–55 years), and 75.0% (56–70 years); this bivariate association was **not** statistically significant (*χ*^2^ = 4.34, *p* = 0.504, Monte Carlo simulation).

Male donors (*n* = 3,234) were 84.0% first-time vs. 61.4% among female donors (*n* = 311) (*χ*^2^ = 37.84, *p* < 0.001).

### 3.3 Temporal trends in donor type (2017–2022)

Table 2 presents yearly donor type distribution. In 2017 and 2018, all recorded donors were first-time donors (100%), though sample sizes were very small (*n* = 2 and *n* = 1, respectively). In 2020, first-time donors constituted 87.7% (1,131/1,290) and repeat donors 12.3% (159/1,290). By 2021, the repeat-donor proportion rose to 19.5% (189/967), and by 2022 to 22.6% (291/1,286). No formal trend test was performed given the small number of years.

**Table 2:** Yearly trends in donor type distribution (2017–2022).

Year	Total *N*	First-time *N* (%)	Repeat *N* (%)
2017	2	2 (100.0)	0 (0.0)
2018	1	1 (100.0)	0 (0.0)
2020	1,290	1,131 (87.7)	159 (12.3)
2021	967	778 (80.5)	189 (19.5)
2022	1,286	995 (77.4)	291 (22.6)

Authors' analysis of blood donor records, 2017–2022. Data for 2017–2018 are based on very small sample sizes; estimates are correspondingly unstable. No donor records for 2019 were available for this analysis [due to unavailable/archived facility registers, a reporting interruption].

### 3.4 Factors associated with first-time donation: univariate analysis

Table 3 presents univariate logistic regression results. Male sex (OR 3.27, 95% CI: 2.55–4.18, *p* < 0.001), non-volunteer donation (OR 3.93, 95% CI: 2.89–5.32, *p* < 0.001), and Oti region relative to Northern (OR 228.83, 95% CI: 87.66–923.30, *p* < 0.001) were associated with higher odds of first-time donation. Increasing age (OR 0.96 per year, 95% CI: 0.95–0.97, *p* < 0.001), urban residence (OR 0.17, 95% CI: 0.14–0.21, *p* < 0.001), Upper West region relative to Northern (OR 0.45, 95% CI: 0.38–0.53, *p* < 0.001), and “Other” region relative to Northern (OR 0.06, 95% CI: 0.04–0.08, *p* < 0.001) were associated with lower odds. Blood group A (OR 1.44, 95% CI: 1.13–1.88, *p* = 0.005) and blood group B (OR 1.28, 95% CI: 1.02–1.62, *p* = 0.033) were associated with higher odds relative to group O; blood group AB (OR 0.55, 95% CI: 0.31–1.04, *p* = 0.052) and Rh positivity (OR 1.28, 95% CI: 0.99–1.63, *p* = 0.054) showed non-significant trends.

**Table 3:** Univariate logistic regression: factors associated with first-time donation.

Variable	OR	95% CI	*P*-value
Male sex	3.27	2.55–4.18	<0.001
Age (per year)	0.96	0.95–0.97	<0.001
Urban residence	0.17	0.14–0.21	<0.001
Non-volunteer donor	3.93	2.89–5.32	<0.001
Blood group A (vs. O)	1.44	1.13–1.88	0.005
Blood group B (vs. O)	1.28	1.02–1.62	0.033
Blood group AB (vs. O)	0.55	0.31–1.04	0.052
Rh positive	1.28	0.99–1.63	0.054
Region: Upper West (vs. Northern)	0.45	0.38–0.53	<0.001
Region: Oti (vs. Northern)	228.83	87.66–923.30	<0.001
Region: Other (vs. Northern)	0.06	0.04–0.08	<0.001

Authors' analysis of blood donor records, 2017–2022.

### 3.5 Factors associated with first-time donation: multivariable analysis

After adjusting for sex, age, residence, donation purpose, and region, age (AOR 0.97 per year, 95% CI: 0.96–0.99, *p* < 0.001) and urban residence (AOR 0.69, 95% CI: 0.49–0.96, *p* = 0.027) remained independently associated with lower odds of first-time donation (Table 4). Male sex (AOR 0.81, 95% CI: 0.59–1.09, *p* = 0.164) and non-volunteer donation (AOR 1.26, 95% CI: 0.90–1.75, *p* = 0.175) were no longer statistically significant. The Upper West region (AOR 4.77, 95% CI: 3.62–6.33, *p* < 0.001) and Oti region (AOR 491.65, 95% CI: 180.34–2,026.31, *p* < 0.001) both had higher adjusted odds relative to Northern; for Upper West, this represents a reversal in direction from the univariate estimate. The Hosmer-Lemeshow goodness-of-fit test indicated acceptable model fit (*χ*^2^ = 6.52, df = 8, *p* = 0.589). The very wide confidence interval around the Oti-region estimate (180.34–2,026.31) signals substantial imprecision, consistent with the small number of repeat-donation events in that region (*n* = 3 of 1,512 donors); this is addressed as a limitation below.

**Table 4:** Multivariable logistic regression: factors associated with first-time donation (adjusted odds ratios).

Variable	AOR	95% CI	*P*-value
Male sex	0.81	0.59–1.09	0.164
Age (per year)	0.97	0.96–0.99	<0.001
Urban residence	0.69	0.49–0.96	0.027
Non-volunteer donor	1.26	0.90–1.75	0.175
Upper West region (vs. Northern)	4.77	3.62–6.33	<0.001
Oti region (vs. Northern)	491.65	180.34–2,026.31	<0.001

Authors' analysis of blood donor records, 2017–2022. Model adjusted for all variables shown. Hosmer-Lemeshow goodness-of-fit test: *χ*^2^ = 6.52, df = 8, *p* = 0.589.

### 3.6 TTI prevalence comparison: first-time vs. Repeat Donors

Table 5 presents TTI prevalence by donor type. First-time donors had higher prevalence of any TTI (i.e., positivity for at least one of HIV, HBV, HCV, or syphilis) than repeat donors (16.06% vs. 5.48%, *χ*^2^ = 48.32, *p* < 0.001), as well as higher HBV prevalence (5.64% vs. 1.88%, *χ*^2^ = 15.73, *p* < 0.001), HCV prevalence (2.51% vs. 0.16%, *χ*^2^ = 14.21, *p* < 0.001), and syphilis prevalence (8.67% vs. 3.60%, *χ*^2^ = 18.82, *p* < 0.001). No HIV infections were detected among repeat donors, while first-time donors had an HIV prevalence of 0.86% (*χ*^2^ = 5.53, *p* = 0.015).

**Table 5:** TTI prevalence by donor type.

Infection	First-time donors (*n* = 2,907)	Repeat donors (*n* = 639)	Chi-square	*P*-value
HIV	25 (0.86%)	0 (0.00%)	5.53	0.015
HBV	164 (5.64%)	12 (1.88%)	15.73	<0.001
HCV	73 (2.51%)	1 (0.16%)	14.21	<0.001
Syphilis	252 (8.67%)	23 (3.60%)	18.82	<0.001
Any TTI	467 (16.06%)	35 (5.48%)	48.32	<0.001

Authors' analysis of blood donor records, 2017–2022.

## 4 Discussion

This analysis provides a comprehensive characterisation of donor type dynamics in Northern Ghana, showing that first-time donors constitute the large majority (82.0%) of the donor population, with substantial geographic and demographic variation. The low proportion of repeat donors (18.0%) represents a blood safety vulnerability, as first-time donors have markedly higher TTI prevalence across all infections examined (3, 4).

### 4.1 Predominance of first-time donors

The finding that 82.0% of donors were first-time donors is broadly consistent with reports from other sub-Saharan African settings where blood donation systems remain under development. In Nigeria, Oyedeji et al. (3) reported first-time donor proportions of up to 85% in a five-year single-centre review. In Burkina Faso, Nagalo et al. (4) documented a similar pattern, with first-time donors comprising approximately 75% of the donor population in a study of 5,423 blood donors. This contrasts with high-income countries, where repeat donors typically constitute 60%–80% of the donor base (1); in the United States, repeat donors account for approximately 73% of donations (21), and in the United Kingdom the repeat-donor rate exceeds 80% (22).

The predominance of first-time donors has direct implications for blood safety, given that first-time donors in this analysis had roughly three times the prevalence of any TTI compared with repeat donors. Several mechanisms plausibly contribute to this pattern. Repeat donors undergo repeated screening and are permanently deferred if they test positive, progressively removing infected individuals from the repeat-donor pool (2, 23). Repeat donors are also more likely to be voluntary, altruistically motivated donors who may have lower baseline risk profiles (1), and they receive recurring pre-donation education that may promote risk-reduction behaviours (24).

### 4.2 Regional disparities in donor type

The regional variation in donor type distribution is striking. The Oti region had an extraordinary 99.8% first-time-donor proportion, with only three repeat donors recorded over six years. The Oti region was created in 2018 (previously part of the Volta Region), and blood donation infrastructure there may still be developing (10); the Nkwanta South Municipal Hospital, which contributed all Oti-region donors, may function largely as a referral centre for emergency family donation with limited opportunity for repeat donation. A comparable pattern has been described by Tagny et al. (25) in their review of blood donors across sub-Saharan Africa, where newly established blood services, including in Cameroon, reported very high first-time-donor proportions.

The Northern region had the highest repeat-donor proportion (29.9%) among the three northern regions studied, plausibly reflecting more established donation infrastructure in Tamale, which hosts three of the study's hospitals. The Upper West region had an intermediate repeat-donor proportion (25.7%). The “Other” region (Techiman) had the highest overall repeat-donor proportion (76.0%), but this estimate should be interpreted cautiously given the small sample from a single facility (179 donors, 5.0% of the total).

These regional disparities suggest that donor retention efforts may be most effective if geographically targeted, with the Oti region a priority for donor recruitment and retention infrastructure and the Northern region potentially informative as a comparator for effective local practices.

### 4.3 Rural-Urban disparities

Rural donors were substantially more likely to be first-time donors (93.1%) than urban donors (69.8%). Plausible contributing factors include lower general access to health facilities, including blood donation services, in rural districts (5), less-developed voluntary donor recruitment infrastructure outside urban centres, with replacement donation predominating in response to a family member's illness (5), and reduced awareness of and access to repeat donation opportunities due to transport and livelihood constraints (25).

Rural donors in this cohort also had higher TTI prevalence than urban donors (syphilis: 11.62% rural vs. 4.16% urban; HCV: 3.99% rural vs. 0.23% urban), which, taken together with the higher proportion of first-time donors in rural areas, compounds the blood-safety burden in these settings. Comparable rural-urban disparities in TTI prevalence have been reported elsewhere in sub-Saharan Africa (25) and specifically in Northern Ethiopia (26).

### 4.4 Donation purpose and donor type

Non-volunteer donors were more likely to be first-time donors (82.5%) than volunteer donors (54.6%) in unadjusted analysis, consistent with the broader evidence base on replacement vs. voluntary donation (1, 2). A systematic review by Piersma et al. (27) found that replacement donors had substantially lower odds of becoming repeat donors compared with voluntary donors. However, in our adjusted analysis, donation purpose was no longer independently associated with donor type after accounting for region and other covariates, suggesting that much of the crude association is explained by geographic factors, replacement donation being more common in rural areas and regions with less-developed voluntary programs.

The very low proportion of voluntary donors overall (5.5% of all donors) is noteworthy. Ghana's national blood collection index rose only modestly between 2016 and 2018 (from 5.8 to 6.0 units per 1,000 population), and the proportion of voluntary unpaid donors nationally remained below approximately 35%–36% over this period (5). Our data suggest that in Northern Ghana specifically, replacement or family-directed donation predominates even more heavily than the national average. Sustained investment in voluntary donor recruitment, including school-based programs, workplace donation drives, and community mobilization, is consistent with WHO guidance on achieving a safe and sustainable blood supply through voluntary donation (1).

### 4.5 Age and sex differences

Younger donors (16–25 years) had the highest proportion of first-time donors (89.4%) in the descriptive data, though this bivariate age-group comparison was not statistically significant (*χ*^2^ = 4.34, *p* = 0.504). In contrast, age modelled as a continuous variable in the multivariable analysis was independently and significantly associated with donor type: each additional year of age reduced the odds of being a first-time donor by approximately 3% (AOR 0.97, 95% CI: 0.96–0.99, *p* < 0.001). This apparent discrepancy is a methodological, not substantive, inconsistency: collapsing a continuous predictor into five uneven categorical strata, two of which were very small (46–55 years, *n* = 136; 56–70 years, *n* = 8), reduces statistical power and can obscure a real, monotonic association that is more efficiently detected when age is modelled continuously and adjusted for confounders (13). Taken together, the multivariable result is the more reliable estimate of the age-donor type relationship and is broadly consistent with U.S. donor-retention literature reporting higher retention among older donor cohorts (24, 28).

The unadjusted association between male sex and first-time donation likely reflects the demographic composition of the donor population (91.2% male); this association did not persist after adjustment (AOR 0.81, 95% CI: 0.59–1.09, *p* = 0.164), suggesting it is substantially explained by other covariates, particularly age and region. This contrasts with studies from high-income countries, where female donors typically show higher retention (24), a difference that may partly reflect cultural and logistical factors affecting women's opportunities to donate in Northern Ghana.

### 4.6 Temporal trends

The increase in repeat-donor proportion from 12.3% in 2020 to 22.6% in 2022 is a positive signal, though it must be interpreted cautiously given the very small 2017–2018 sample sizes and the absence of 2019 data. Several non-mutually-exclusive explanations are plausible: donors who began donating during COVID-19-related shortages may have continued donating afterward (29, 30); National Blood Service Ghana donor-retention initiatives, including SMS recall systems, may be gaining traction (2); and facility-level record-keeping may have improved over the period, making repeat donors easier to identify. The 2022 repeat-donor proportion (22.6%) nonetheless remains well below the WHO-referenced target range of 60%–80% repeat donors for a stable, safe blood supply (1). Continued monitoring over subsequent years is needed to determine whether this trend is sustained.

### 4.7 TTI burden in first-time donors

The substantially higher TTI prevalence among first-time donors (16.06% vs. 5.48%) is consistent with findings from other African countries. In Northern Ethiopia, Legese et al. (14) reported that first-time donors had markedly higher odds of HBV infection compared with repeat donors. In Nigeria, Oyedeji et al. (3) similarly documented that first-time donors accounted for most TTI-positive donations in their Lagos cohort. In Burkina Faso, Nagalo et al. (4) found first-time donors had significantly higher prevalence of HIV, HBV, and syphilis than repeat donors.

The low TTI prevalence among repeat donors likely reflects, at least in part, the effectiveness of repeated screening and deferral in removing infected individuals from the repeat-donor pool; the absence of any HIV infections among repeat donors is consistent with effective identification and deferral under national guidelines (2). These findings support prioritising enhanced pre-donation screening and health education for first-time donors, and support the programmatic value of converting first-time donors into repeat donors as early as feasible, given the lower TTI burden this group appears to carry over time.

### 4.8 Comparison with other studies

The repeat-donor proportion in our study (18.0%) is lower than reported in more established African blood services, approximately 60% in South Africa (31) and 30%–40% in Kenya (32), and is more comparable to Nigeria (15%–25% in most regions) (3) and Cameroon (78% first-time donors, i.e., 22% repeat) (25). The regional heterogeneity observed within our own data (0.2% repeat donors in Oti vs. 76% in the “Other” region) illustrates that national or even regional averages can mask substantial local variation, underscoring the value of facility- or district-level data for planning donor retention interventions.

### 4.9 Study limitations

Several limitations warrant consideration. First, donor-type classification relied on single-facility records and could not capture donations made elsewhere; a donor classified as “first-time” at one facility may have donated previously at another, which would bias findings toward underestimating the true proportion of repeat donors (9). Second, the study period included the COVID-19 pandemic years, which may have affected donation patterns in ways not generalisable to other periods (30). Third, the very small 2017–2018 samples (*n* = 2 and *n* = 1) limit the reliability of early trend estimates, and the absence of 2019 data further limits interpretation of the temporal trend. Fourth, data on reasons for non-return (deferral, illness, relocation, death) were unavailable, limiting insight into specific barriers to repeat donation. Fifth, donor motivation was captured only as a binary volunteer/non-volunteer classification, without finer-grained data on altruism or social pressure. Sixth, the RDTs used for TTI screening have both lower sensitivity and lower specificity than laboratory-based assays (e.g., ELISA or nucleic acid testing); lower sensitivity may have led to underestimation of true TTI prevalence, while lower specificity could inflate apparent prevalence through false-positive reactions, although the latter risk is partly mitigated by the confirmatory second RDT of a different brand used in this study. Seventh, the blood group analysis was exploratory, was not adjusted for multiple comparisons, and should be interpreted as hypothesis-generating rather than confirmatory. Eighth, findings may not generalise to southern Ghana (e.g., Greater Accra, Ashanti), where donor populations and health infrastructure differ from the northern, largely rural and semi-urban settings studied here. Ninth, the adjusted odds ratio for the Oti region (AOR 491.65, 95% CI: 180.34–2026.31) is exceptionally large and imprecise, reflecting the very small number of repeat-donation events in that region (*n* = 3 of 1,512 donors, 0.2%); once this count is further partitioned across the covariates in the multivariable model, several covariate combinations likely contained few or no repeat-donor events, a sparse-data condition that can produce quasi-complete separation and inflate both the point estimate and its confidence interval (17, 18). We recommend that future analyses of these or similar sparse regional strata apply Firth's or an equivalent penalized-likelihood method directly within the multivariable model.

Despite these limitations, the overall sample size and inclusion of multiple facilities across three regions provide a robust basis for the observed patterns.

### 4.10 Policy and program implications for donor retention

These findings suggest several specific priorities for blood safety programs in Ghana, which should be considered alongside the retrospective, single-region design of this study:

1. The Oti region would benefit from priority investment in donor retention infrastructure, including establishing a donor database, implementing SMS recall systems, training staff in donor retention strategies, and community education on repeat donation (1).
2. With 93.1% of rural donors being first-time donors, rural-focused retention programs warrant consideration. Mobile blood donation services returning to the same communities at regular intervals could help rural donors transition to repeat status, with community health workers supporting recall and education (25).
3. Voluntary donors (5.5% of the total) had substantially higher repeat-donation rates (45.4% vs. 17.5% among non-volunteer donors), supporting programs that convert replacement donors to voluntary donors, including acknowledging replacement donors' contribution and inviting them to become regular voluntary donors (2).
4. Young donors (16–25 years) made up 41.1% of the donor population but had comparatively low repeat-donation rates (10.6%). School-based blood donation programs emphasising lifelong, regular donation could help establish repeat-donation habits early (24).
5. Age-appropriate retention strategies may be worth exploring, as older and younger donors may respond to different motivators (e.g., health check-ups vs. social recognition) (15).
6. The Northern region (29.9% repeat donors) and the “Other” region (76.0% repeat donors) achieved comparatively higher repeat-donation rates. Documenting the specific strategies used in these settings and assessing their transferability to the Oti and Upper West regions may help accelerate improvement elsewhere.

## 5 Conclusions

This analysis of 3,546 blood donors in Northern Ghana found that first-time donors constitute 82.0% of the donor population, while repeat donors comprise 18.0%. The proportion of repeat donors increased from 12.3% in 2020 to 22.6% in 2022, suggesting that donor retention efforts may be gaining traction, although the overall repeat-donation rate remains low relative to WHO-referenced targets. Rural donors (93.1% first-time) and the Oti region (99.8% first-time) represent priority populations for donor retention interventions. First-time donors had substantially higher TTI prevalence across all infections examined, with 16.06% testing positive for at least one TTI compared with 5.48% of repeat donors. These findings support the value of targeted donor retention programs, particularly in rural areas and regions with low repeat-donation rates, as one component of efforts to strengthen blood safety in Northern Ghana.

## Data Availability

The original contributions presented in the study are included in the article/Supplementary Material, further inquiries can be directed to the corresponding author.
