# Supplementary material for: Donor type dynamics and associated factors among blood donors in northern Ghana: a six-year retrospective analysis
Source: Front Epidemiol. 2026 Sep 8;6:1905551. doi: 10.3389/fepid.2026.1905551 (PMC13593756; doi:10.3389/fepid.2026.1905551)
Supplement: Supplementary file 1 [file Table1.docx]

**Supplementary Materials**

**Supplementary Table S1. Overall donor type distribution**

| Donor type | N | Percentage (%) |
| --- | --- | --- |
| First time | 2,907 | 82.0 |
| Repeat | 639 | 18.0 |
| **Total** | **3,546** | **100.0** |

**Supplementary Figure S1. Yearly trend in donor type distribution (2017-2022)**

**Supplementary Figure S2. Donor type distribution by region**

**Supplementary Table S2. Donor type by blood group**

| Blood group | First-time N (%) | Repeat N (%) | Total N |
| --- | --- | --- | --- |
| A | 489 (85.5) | 83 (14.5) | 572 |
| AB | 42 (79.2) | 11 (20.8) | 53 |
| B | 580 (84.7) | 105 (15.3) | 685 |
| O | 1,755 (78.7) | 474 (21.3) | 2,229 |

**Supplementary Table S3. TTI prevalence by donor type**

| Infection | First-time donors (n=2,907) | Repeat donors (n=639) | Chi-square | P-value |
| --- | --- | --- | --- | --- |
| HIV | 25 (0.86%) | 0 (0.00%) | 5.53 | 0.015 |
| HBV | 164 (5.64%) | 12 (1.88%) | 15.73 | <0.001 |
| HCV | 73 (2.51%) | 1 (0.16%) | 14.21 | <0.001 |
| Syphilis | 252 (8.67%) | 23 (3.60%) | 18.82 | <0.001 |
| Any TTI | 467 (16.06%) | 35 (5.48%) | 48.32 | <0.001 |

**Supplementary Figure S3. TTI prevalence comparison by donor type**

**Supplementary Table S4. Donor type by Rh status**

| Rh status | First-time N (%) | Repeat N (%) | Total N |
| --- | --- | --- | --- |
| Negative | 340 (78.9) | 91 (21.1) | 431 |
| Positive | 2,561 (82.4) | 548 (17.6) | 3,109 |
